# Identifying lower limb specific and generalised joint hypermobility in adults: validation of the Lower Limb Assessment Score

**DOI:** 10.1186/s12891-017-1875-8

**Published:** 2017-12-06

**Authors:** Kaitlin J. Meyer, Cliffton Chan, Luke Hopper, Leslie L. Nicholson

**Affiliations:** 1 Discipline of Physiotherapy, Faculty of Health Sciences, The University of Sydney, Sydney, New South Wales Australia; 2 Discipline of Biomedical Science, Sydney Medical School, The University of Sydney, Sydney, New South Wales Australia; 3 Western Australian Academy of Performing Arts, Edith Cowan University, Perth, Western Australia Australia

**Keywords:** Joint hypermobility, Joint instability, Joint laxity, Lower extremity, Screening tool, Manual tests, Orthopaedic tests

## Abstract

**Background:**

The Lower Limb Assessment Score (LLAS) has only been validated in a paediatric population. The aim of this study was to validate the use of the LLAS in an adult population by: i) evaluating its ability to discriminate between different extents of lower limb hypermobility, ii) establishing a cut-off score to identify lower limb hypermobility, and iii) determining if the LLAS is able to identify Generalised Joint Hypermobility (GJH).

**Methods:**

Participants were recruited across three groups representing varying degrees of hypermobility. They were assessed using the LLAS, Beighton score and clinical opinion. Pearson’s correlation coefficient and MANOVA were used to assess between-group differences in the LLAS. The cut-off score was determined using median and inter-quartile ranges and the Receiver Operator Characteristic Curve. The ability of the LLAS to identify GJH was assessed using percent agreement with clinical opinion.

**Results:**

One hundred twelve participants aged 18–40 years were recruited. The LLAS distinguished the control from the likely hypermobile and known hypermobile cohorts (both *p* < 0.001), as well as the likely hypermobile from the known hypermobile cohort (*p* = 0.003). The LLAS cut-off score for identifying lower limb hypermobility was ≥7/12 with a specificity of 86% and sensitivity of 68%. The LLAS accurately identified those with GJH with high percentage agreement compared to clinical opinion across all cohorts (69–98%).

**Conclusions:**

The LLAS is a valid tool for identifying lower limb specific hypermobility and GJH in adults at a cut-off score of ≥7/12. It demonstrates excellent specificity and moderate sensitivity, and discriminates well between extents of hypermobility.

**Electronic supplementary material:**

The online version of this article (10.1186/s12891-017-1875-8) contains supplementary material, which is available to authorized users.

## Background

Hypermobility is present when a joint’s active or passive range exceeds that expected for an individual’s age, gender and ethnicity [1]. To be truly hypermobile, the range should, by definition, be in the uppermost 5% of the population [2]. Hypermobility may be localised to select joints or present in multiple body areas (typically greater than five sites) as Generalised Joint Hypermobility (GJH) [1]. In addition to the influence of age, gender and ethnicity, joint specific hypermobility can be acquired through regular training or environmental factors such as trauma or surgery, and can also be inherited [1, 3].

The presence of GJH can be asymptomatic, or conversely be associated with local or widespread musculoskeletal problems [4, 5]. Hypermobile joints may be more prone to macrotrauma (such as dislocations or soft tissue injuries) resulting in acute pain and reduced function, as well as microtrauma, which may predispose the individual to recurrent musculoskeletal pain and early joint degeneration [1]. Further, joint hypermobility can be a marker of various heritable connective tissue disorders including but not limited to hypermobile Ehlers-Danlos Syndrome (hEDS), previously known as both Joint Hypermobility Syndrome (JHS) and Ehlers-Danlos Syndrome Hypermobility Type (EDS-HT) [1, 6]. However the assessments for joint hypermobility have largely focussed on paediatric populations and a paucity of evidence is available to direct clinical evaluation of such hypermobility in adult populations.

There is a wide variation in the reported prevalence of GJH and hEDS. The population prevalence of GJH is estimated to be 10–30%, with significantly higher rates amongst children, females, and Asian and African racial groups [7, 8]. A reduction in joint mobility is also demonstrated with age [3]. A minimum estimate of 1 in 5000 has previously been proposed for EDS, of which hEDS is likely to account for 80–90% of cases [9, 10]. However, epidemiologic studies determining an accurate figure for population prevalence of the condition have yet to be performed [11].

The Beighton score is the most widely used tool for identifying GJH and plays a significant role in the diagnosis of hEDS [6, 12]. Despite its utility as a simple epidemiological clinical tool, the Beighton score is dominated by tests of the upper extremity, with knee extension being the only lower limb specific inclusion [13]. In addition it does not include several body sites that are commonly and symptomatically hypermobile, such as the hips, ankles, and first metatarsophalangeal joints [5]. Consequently, the Beighton score may not validly identify generalised hypermobility when it presents predominantly in the lower limbs [13, 14].

The Lower Limb Assessment Score (LLAS) was designed to provide greater levels of detail on lower limb hypermobility by assessing both accessory and physiological range of motion of the hip, knee, ankle and rear, mid and forefoot [13]. It has demonstrated excellent intra-rater and inter-rater reliability [13, 15], and a validated cut-off score of ≥7/12 to identify lower limb hypermobility in children [13]. Ferrari and colleagues’ concluding recommendations were to reproduce the study in older ages and different ethnicities to account for the wide variation in sensitivity and specificity across groups [13].

The primary aim of this study was to determine the validity of the LLAS in identifying lower limb hypermobility in an adult population. This was achieved by: i) evaluating the ability of the LLAS to discriminate between normal mobile individuals, those with likely hypermobility, and those with symptomatic hypermobility and ii) determining a cut-off score for the identification of lower limb hypermobility in adults when using the LLAS. The secondary aim of the study was to assess if the LLAS could be used to identify GJH, or only hypermobility of the lower limbs.

## Methods

The method for this study was based on the protocol employed by Ferrari and colleagues (2005) who validated the LLAS clinical tool in a school-aged population (5–16 years).

### Participants

A total of 112 participants were recruited across three groups that were selected to represent varying levels of joint hypermobility. They comprised people with: i) normal mobility (controls), ii) likely hypermobility and iii) known symptomatic hypermobility. The control group were healthy individuals who had not undertaken long-term training likely to affect joint mobility such as dance, gymnastics and acrobatics. These participants were recruited from the University of Sydney Cumberland Campus using flyers on noticeboards. The likely hypermobile group were comprised of elite dancers who were either studying dance at tertiary level with a majority of their training in ballet or who were currently dancing professionally (working as a dancer full-time). They were recruited via flyers, emails, and verbal invitations by a liaison officer from the Western Australian Academy of Performing Arts (Edith Cowan University) and the West Australian Ballet (Perth). The known hypermobile group were people with a prior medical diagnosis of JHS/EDS-HT under the previous nosology, and were recruited from Australian JHS/EDS-HT patient support groups on Facebook. For all groups, participants between the ages of 18–40 years of either gender were included. Volunteers older than 40 years were excluded due to the possibility that joint range could be affected by degenerative disease [3]. Participants across all groups were also excluded if diagnosed with another hereditary disorder of connective tissue (e.g. Marfan syndrome, osteogenesis imperfecta or other subtypes of EDS), or if they had a current orthopaedic condition that might restrict normal range of motion.

All involvement was voluntary and participants provided written informed consent prior to data collection. Ethical approval was gained from the Sydney University [Protocol No. 2015/465] and Edith Cowan University [Protocol No. 13174] Human Research Ethics Committees.

### Procedure

A survey was used to collect information on age, gender, ethnicity, clinical history, current presentation and self-perceived hypermobility. All participants attended a single physical testing session where they were assessed using i) the LLAS (Additional file 1) to provide a maximum score out of 12 for each limb [13] and ii) the Beighton Score (Additional file 2), providing a single score out of 9 [3]. All assessments were completed in the same order without prior warm up and were conducted by experienced physiotherapists (CC and LN) with over 30 years of combined clinical expertise in the domains of sports and musculoskeletal physiotherapy. Each participant was also categorised as hypermobile, borderline hypermobile or normal according to the clinical opinion of these physiotherapists. Given there remains some subjectivity in the sole use of the Beighton score for determining GJH, expert clinical opinion was used [1]. This clinical judgment was based on a comprehensive history, the validated five-part historical hypermobility questionnaire [16], Beighton score, and the physical assessment of peripheral joints, not using the LLAS. Cut-off scores of ≥4/9 and ≥5/9 on the Beighton score were used for men and women respectively in the control and known hypermobile cohorts [2]. A higher score of ≥6/9 was considered GJH in the likely hypermobile cohort to account for the debated palms to floor measure in dancers [17, 18].

In addition, to determine the inter-rater reliability of the LLAS assessments the data from the first 20 control and known hypermobile participants assessed by both examiners were analysed. Given that the objective was to determine whether the LLAS is a robust measure for clinical practice, intra-rater reliability was not of major concern for this study. Furthermore, logistically it was not possible for many of the EDS-HT/JHS participants to return for a second testing session. The inter-rater reliability was assessed using a two-way random absolute agreement, single measures intraclass correlation coefficient. This revealed good to excellent inter-rater reliability (ICC_2,1_ = 0.85, 95% CI = 0.67 to 0.94, *p* < 0.001) [19]. Given the small sample size, a Bland and Altman plot was also used similarly demonstrating no significant difference between the results of the two examiners (limits of agreement = −2.94 and 2.44, 95% C.I. = −0.89 to 0.39, *p* = 0.43). Therefore, a single examiner was used to assess the remaining participants.

### Statistical analysis

Statistical analysis of collected data was performed using SPSS Version 21 (IBM, NY, USA). The data were inspected for outliers and tested for normality using calculations of skewness. Descriptive statistical analysis was performed on age, gender and ethnicity. Pearson’s correlation coefficient was used to test the association between the LLAS and age. Pearson’s (*r*) correlations of ≤0.35 were considered to be weak, 0.36–0.67 moderate, and ≥0.68 strong [20]. A paired samples t-test was used to compare the LLAS scores obtained from the left and right limbs.

To test the null hypothesis of no significant difference being detectable between the three groups when using the LLAS, a multivariate analysis of variance (MANOVA) was performed, followed by post-hoc analyses (Tukey HSD) to reveal where the significant differences occurred between the three groups. Effect sizes (partial η^2^) greater than 0.01 were considered small differences, greater than 0.06 were considered medium differences, and values greater than 0.14 were representative of a large difference between groups [21].

The cut-off score to represent hypermobility when using the LLAS was estimated using median and inter-quartile ranges for each group. The sensitivity and specificity for each level of the LLAS was tested using Receiver Operator Characteristic (ROC) curve analysis. A cut-off point that maximised specificity whilst optimising sensitivity was selected in order to minimise false positive errors [22]. The positive and negative likelihood ratios were calculated for the determined cut-off point, combining the utility of sensitivity and specificity [22]. Clinical opinion was used as the gold standard for this analysis, and the adults who were initially categorised as “borderline hypermobile,” were reclassified as normal [13]. This was based on the decision that if the clinician was uncertain of the classification, then the participant was less likely to be clinically hypermobile. Finally, the percent agreement between the LLAS (using the newly established cut-off point) and clinical opinion was calculated to determine if it could be used to accurately predict GJH. Clinical opinion was used as the gold standard for the identification of GJH.

For this study, a *p*-value <0.05 was considered significant. It was powered at 90% to minimise the possibility of type 2 errors, with α at 2.5% (type 1 error rate). The probability of GJH in the general population ranges from 15 to 30% [7, 8] so an average would be 15% (i.e. 0.15 pA), whilst the probability of hypermobility in the experimental cohort (likely hypermobile and diagnosed JHS/EDS-HS groups combined) is high, with a conservative estimate of 60% (i.e. 0.6 pB). Therefore, a minimum of 31 controls and 62 hypermobile participants (31 in likely and 31 in known) were needed to be able to reject the null hypothesis that the LLAS is equivocal in both groups at this level of power (PS Power and Sample Size Calculations Version 3.0, 2009).

## Results

General participant demographics are summarised in Table 1. The participants across each cohort were predominantly Caucasian, female and between 20 and 30 years of age. The known hypermobile group was significantly older than both the controls (*p* < 0.001) and the likely hypermobile group (*p* < 0.001). Weak associations were found between the LLAS total score with age across the three groups (controls: *r* = −0.20; likely hypermobiles: *r* = 0.23; known hypermobiles: *r* = −0.05).

**Table 1:** Demographic data of the entire cohort and the three subgroups

	Total	Controls	Likely Hypermobiles	Known Hypermobiles
	*n = 112*	*n = 40*	*n = 40*	*n = 32*
*Gender n (%)*				
Males	28 (25%)	11 (28%)	16 (40%)	1 (3%)
Females	84 (75%)	29 (72%)	24 (60%)	31 (97%)
*Age (years)*				
Mean ± SD	24.3 ± 5.5	22.6 ± 2.8	22.6 ± 4.4	28.4 ± 6.9
Range	18–40	19–32	18–33	18–40
*Ethnicity n (%)*				
Caucasian	88 (79%)	24 (60%)	34 (85%)	30 (94%)
Asian	16 (14%)	12 (30%)	4 (10%)	0 (0%)
Other	8 (7%)	4 (10%)	2 (5%)	2 (6%)

The means and standard deviations of the LLAS revealed no differences between the left (L) and right (R) sided scores in the controls (L: 3.48 ± 1.95, R: 3.55 ± 1.91, *p* = 0.67) and the known hypermobile cohort (L: 6.91 ± 1.44, R: 6.91 ± 1.87, *p* = 1.0). A statistically significant difference in side-to-side LLAS was found in the likely hypermobile cohort (L: 5.43 ± 2.01, R: 6.15 ± 2.26, *p* = 0.01). Whilst there was a higher average score on the right compared to the left side in the side in this group, there was also a high correlation between the two scores (*r* = 0.73). Therefore, the LLAS was recorded as a score out of 12 for a randomly chosen limb, rather than the total of 24 for both limbs of all participants.

### Using the LLAS to discriminate between extents of hypermobility

**Fig. 1:**
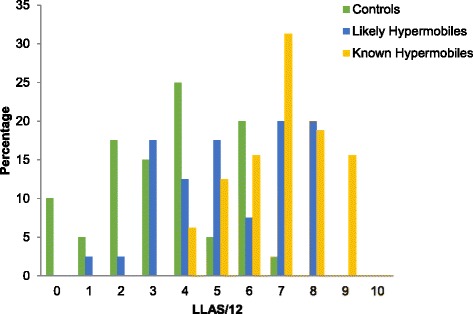
Percentage of participants under each category/score of the Lower Limb Assessment Score (LLAS)

The percentage of adults at each level of the LLAS (total score/12) was examined (Fig. 1).

Observation of the distribution of scores in Fig. 1 suggests that the LLAS was able to discriminate clearly between all groups, with overlap mostly at scores of four and six out of 12. The MANOVA confirmed a statistically significant difference between the three groups when assessed using the LLAS (Pillai’s Trace = 0.45, F(4.0, 218.0) = 15.8, *p* < 0.001). There was a large difference between the groups with an effect size (partial η^2^) of 0.23. The post-hoc Tukey analysis revealed statistically significant mean differences in LLAS scores between all three groups: comparing the controls to the likely hypermobile cohort (MD = −1.95, 95% CI = −2.93 to −0.97, *p* < 0.001) and to the known hypermobile cohort (MD = −3.43, 95% CI = −4.47 to −2.39, *p* < 0.001), and comparing the likely hypermobile cohort to the known hypermobile cohort (MD = −1.48, 95% CI = −2.52 to −0.44, *p* = 0.003).

### Establishing a cut-off score for the identification of lower limb hypermobility

**Fig. 2:**
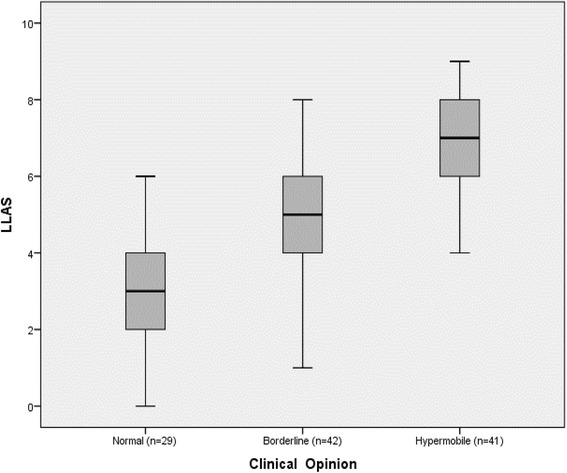
Median and interquartile ranges of the Lower Limb Assessment Score (LLAS) across the clinically defined groups

**Fig. 3:**
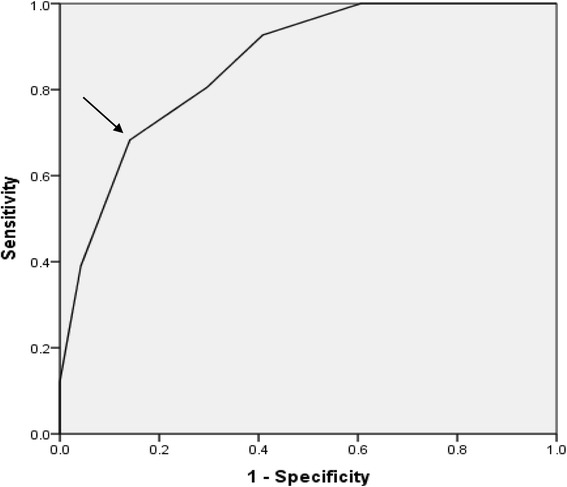
Receiver Operator Characteristic Curve for the Lower Limb Assessment Score

The median and interquartile ranges for the LLAS across the three clinically defined groups are represented in Fig. 2. This figure suggests that a cut-off score of seven would clearly differentiate the controls from the known hypermobiles, with minimal overlap with the borderline individuals. This observation was confirmed by the ROC Curve presented in Fig. 3. The most suitable cut-off score was seven, where sensitivity was 0.68 and specificity was 0.86 (Table 2). This produced a positive likelihood ratio of 4.84 and a negative likelihood ratio of 0.37.

**Table 2:** Sensitivity and 1-specificity for items of the Lower Limb Assessment Score

Cut-Off	Sensitivity	1-Specificity
0	1.000	1.000
1	1.000	0.944
2	1.000	0.901
3	1.000	0.789
4	1.000	0.606
5	0.927	0.408
6	0.805	0.296
**7** ^a^	**0.683**	**0.141**
8	0.390	0.042
9	0.122	0.000
10	0.000	0.000

^a^The sensitivity and 1-specificity for the decided cut-off point of 7/12 are in bold for distinction

Given the predominance of Caucasian individuals in the study’s cohort, the same analysis was repeated for the 88 Caucasian participants. The most suitable cut-off score for this group was seven, with a sensitivity of 0.68 and specificity of 0.84.

### Comparing the lower limb assessment score with clinical opinion: Can the LLAS and be used to accurately identify GJH?

Having identified a cut-off score of ≥7/12 for the identification of lower limb hypermobility, the LLAS identified lower limb hypermobility in one out of the 40 (3%) control, 16/40 (40%) likely hypermobile and 21/32 (66%) known hypermobile participants. High levels of agreement were found between the LLAS and clinical opinion across each cohort, especially in the control group at 98% agreement, followed by the likely hypermobile group at 70% and the known hypermobile group at 69% (Table 3).

**Table 3:** Cross tabulations of the agreement between the Lower Limb Assessment Score (LLAS) and clinical opinion across the three cohorts

		Clinical Opinion		
Cohort	LLAS	Not HM^b^	HM	Total
*Controls*	Not HM	38	1	39
	HM	0	1	1
	*Total*	*38*	*2*	*40*
*Likely HMPs* ^a^	Not HM	22	2	24
	HM	10	6	16
	*Total*	*32*	*8*	*40*
*Known HMPs*	Not HM	1	10	11
	HM	0	21	21
	*Total*	*1*	*31*	*32*

^a^HMPs = hypermobile participants

^b^HM = hypermobile

## Discussion

The Lower Limb Assessment Score was found to be a valid and clinically useful screening and assessment tool, with excellent inter-rater reliability, for the identification of lower limb hypermobility and GJH. A cut-off point of ≥7/12 was determined to be the optimal classification point for the whole cohort, with a high specificity (86%), moderate sensitivity (68%) and positive and negative likelihood ratios of 4.84 and 0.37 respectively. The positive likelihood ratio as it is greater than three, and the negative likelihood ratio as it is close to one-third, are likely to be useful in determining an accurate post-test probability of hypermobility when using the LLAS in a clinical setting [23].

With a predominance of Caucasian participants in the study and the potentially confounding effect of different ethnicities, the analysis was repeated with the 88 Caucasian participants. This revealed an identical cut-off score of ≥7/12, with a high specificity (84%) and moderate sensitivity (68%). It is expected that a higher cut-off point may be found in a wholly non-Caucasian population due to the increase in hypermobility demonstrated in Asian and African racial groups [8]. However, these results remain applicable to an Australian population.

It was hypothesised that the cut-off score for adults would be lower than the ≥7/12 proposed by Ferrari and colleagues (2005) for children due to the reduction of hypermobility seen with age [3, 13]. Whilst this study found an equal score to that of the pre-pubescent paediatric cohort, this may have been a reflection of the individuals included in this study, with 75% female participants, over half being 22 years of age or younger. Given these factors are known to increase the prevalence of hypermobility [8, 24], this may have contributed to the higher cut-off score than expected in this younger female-dominant adult population. Although the participants in this study were predominantly young adults with a mean age of 24.3 years, the cohort was intentionally restricted to participants younger than 40 to exclude greater hypomobility as a result of joint degeneration.

The LLAS, as a continuous measure, was able to discriminate between varying extents of hypermobility. No studies have investigated this in adults, but the findings in this study are consistent with the results found when using the LLAS in a paediatric population [13]. Although only weak associations were found between the LLAS and age, the control cohort demonstrated the expected reduction in hypermobility seen with older ages whilst the likely hypermobile cohort demonstrated an increase in hypermobility. This is possibly a result of acquired lower limb hypermobility from classical dance training amongst this cohort [17, 25, 26]. The known hypermobile cohort, despite being significantly older than both the controls and the likely hypermobiles, demonstrated almost no association between the LLAS and age. This suggests less “stiffening” with age when compared to the controls, however currently there is limited prospective research into the natural history of hypermobility in individuals for comparison [1].

The final comparison of the LLAS as a dichotomous screening tool with clinical opinion demonstrated its usefulness in identifying GJH, and not solely hypermobility of the lower limb. A positive LLAS appeared to reflect correct identification of GJH, which was supported by the high level of agreement demonstrated in the control cohort (98%), and the good level of agreement in the likely hypermobile cohort (70%) and in the known hypermobile cohort (69%). In the likely hypermobile group, 10 participants had a positive LLAS which was not reflected by the clinical opinion. This was unsurprising given the potential impact of classical dance training on hypermobility of the lower limb, which was confirmed by high LLAS scores amongst this cohort [17, 25, 26]. In the known hypermobile group, 10 cases of GJH identified by clinical opinion were not identified by the LLAS. It is possible that these individuals presented with hypermobility predominantly of the upper limbs which would go undetected using the LLAS. This supports the importance of incorporating clinical judgement into the identification of GJH, particularly in the hEDS cohort, rather than using the results of a single assessment tool [6].

This study supports the use of the LLAS as a clinical screening and assessment tool in aiding the identification of lower limb hypermobility and GJH at a cut-off point ≥7/12. The clinical utility of this tool over the Beighton score is evident when patients present with predominantly lower limb instability symptoms or are participating in activities that are likely to involve physiological or non-physiological movements to the ends of joint range. It allows identification of hypermobility in lower limb joints commonly predisposed to injury and in directions unassessed by the Beighton score. [5]. An example of the clinical utility of the LLAS in targeting prevention and management is where a ballet dancer might demonstrate limited external rotation at the hip, executing turn-out primarily from knees hypermobile in the transverse plane. The clinical cognisant of this will consider management directed at dissipating rotational forces throughout the limb to reduce injury risk at the knee. The comprehensive nature of the LLAS scores can then be utilised to identify GJH, prompting further assessment of other joints of the body if required. Although the 12 tests take longer to complete than the more commonly used Beighton score, the significant correlation between the left and right sided scores in the control and known hypermobile cohorts indicate that the LLAS could be administered on a single limb to maximise time efficiency. However, the side-to-side differences in our dancer cohort suggest the LLAS should still be performed on both sides for this population.

### Limitations and directions for future research

Difficulties arise when validating the LLAS due to the lack of a gold standard for GJH classification. While the Beighton score has gained international acceptance for the identification of GJH, the variation in cut-off scores used and the lack of a standardised protocol limits the comparability across reliability studies [27, 28]. Clinical opinion was therefore used in this study as the most appropriate alternative for comparison, however future research into a standardised investigative procedure for GJH is warranted.

The generalisability of the cut-off score in this study is limited to a predominantly young female and Caucasian adult population. A higher proportion of male participants and a higher mean age would also be useful to more accurately reflect the older adult population. Additionally, this study was not designed to investigate the LLAS cut-off scores in differing ethnicities. Therefore investigation into the use of the LLAS across varying racial groups would be beneficial. Further, whilst the recruitment across three groups in this study was not equal as intended, the sample size allowed the study to remain sufficiently powered to detect between group differences.

Future research into the clinimetric properties of the LLAS would be of value to determine which of the 12 tests are clinically most useful, and might assist the revision of the LLAS into a more efficient assessment tool. Finally, as the examiners in this study are experienced musculoskeletal physiotherapists, the inter-rater reliability of the LLAS when implemented by less experienced assessors should be investigated.

## Conclusions

In an adult population, the LLAS is a valid tool for identifying lower limb specific hypermobility and GJH at a cut-off point of ≥7/12. It demonstrates excellent specificity, moderate sensitivity, and discriminates well between extents of hypermobility. The increased level of detail on lower limb specific hypermobility provided by the LLAS can assist in the development of targeted prevention and management strategies for at-risk populations such as dancers or gymnasts, as well as for those with symptomatic hypermobility, by highlighting the problematic areas for the individual and allowing those to be addressed accordingly. This identification may assist in both the prevention as well as appropriate treatment of hypermobility related injuries in the lower limb. However, it is recommended that clinical judgment is also incorporated into the identification of GJH, particularly within the hEDS cohorts.

## Additional files

Additional file 1: The Lower Limb Assessment Score. This file contains the name, patient and therapist instructions and criteria for each of the 12 tests of the Lower Limb Assessment score as described by the original authors that developed this tool. (DOCX 17 kb)

Additional file 2: The Beighton Score. This file contains the standardised criteria of the five tests involved in scoring of the Beighton in this study. (DOCX 19 kb)

## Abbreviations

EDS-HT: Ehlers-Danlos Syndrome Hypermobility Type
GJH: Generalised Joint Hypermobility
hEDS: hypermobile Ehlers-Danlos Synrome
JHS: Joint Hypermobility Syndrome
JHS/EDS-HT: Joint Hypermobility Syndrome/Ehlers-Danlos Syndrome Hypermobility Type
LLAS: Lower Limb Assessment Score
MANOVA: Multivariate analysis of variances
ROC: Receiver operator characteristic
