# Supplementary material for: Identifying lower limb specific and generalised joint hypermobility in adults: validation of the Lower Limb Assessment Score
Source: BMC Musculoskelet Disord. 2017 Dec 6;18:514. doi: 10.1186/s12891-017-1875-8 (PMC5719901; doi:10.1186/s12891-017-1875-8)
Supplement: Supplementary file 1 — The Lower Limb Assessment Score. This file contains the name, patient and therapist instructions and criteria for each of the 12 tests of the Lower Limb Assessment score as described by the original authors that developed this tool. (DOCX 17 kb) [file 12891_2017_1875_MOESM1_ESM.docx]

# **ADDITIONAL FILE 1**

The Lower Limb Assessment Score [13]

|  | LEFT | | RIGHT | |
| --- | --- | --- | --- | --- |
| **HIP FLEXION** The patient lies supine; the examiner flexes one hip fully; the other leg must stay fully extended on the couch.  Does the mid-anterior area of the thigh drop easily onto the stomach/chest with a loose feel to the movement, using a minimum to moderate application of force? | YES | NO | YES | NO |
| **HIP ABDUCTION** The patient lies supine, with hip and knees flexed; the knees are dropped outwards and down to the couch, the soles of the feet remain together.  With the examiner’s hand against the lateral femoral condyle, can the knees come down to the couch sufficiently to let the back of the examiner’s hand touch the couch? | YES | NO | YES | NO |
| **KNEE HYPEREXTENSION** The patient lies supine; the knees are relaxed and straight, with minimal force, keeping the femoral condyles on the couch, can the heel be lifted at least 3cm off the couch (greater than 2 finger widths)? | YES | NO | YES | NO |
| **KNEE ANTERIOR DRAWER TEST** The patient is supine; the hips and knees (90°) are flexed; the examiner gently sits on the foot to stabilise it; moderate pressure is placed against the femoral condyles as the tibia is pulled forwards.  Is there a definite, obvious forward movement of the tibia against the femur? Palpable “clunking” of the joint surfaces moving against each is indicative of a positive draw sign. | YES | NO | YES | NO |
| **KNEE ROTATION** The patient lies supine; the examiner flexes the hip and knee to 90° and palpates the tibial tubercle; holding the malleoli and ankle firmly, the tibia is rotated medially and laterally on the femur.  Normal movement is 1cm medially and laterally. Does the tubercle move easily beyond 1cm in any direction or greater than 2cm overall?  With increased internal movement the head of the fibula/lateral condyle of the tibia may also be seen to move. | YES | NO | YES | NO |
| **ANKLE JOINT DORSIFLEXION** The patient lies supine; the knee is flexed to 45°; with moderate to strong force the ankle is dorsiflexed. Does the ankle flex more than 15 degrees?  Along with the increased movement there may be bulging of the skin and subcutaneous fat anterior to the ankle. | YES | NO | YES | NO |
| **ANKLE ANTERIOR DRAW TEST** The patient lies supine; the knee is flexed to 45°; the examiner grasps the heel along the plantar and posterior surfaces with one hand and applies a stabilising force against the anterior of the tibia with the other hand.  Using a strong anterior force, can the calcaneum and talus be brought forwards on the tibia?  Any forward movement felt is a positive result. | YES | NO | YES | NO |
| **SUBTALAR JOINT INVERSION** The patient is supine with their feet over the end of the couch; the examiner holds the posterior surface of the heel and moves the heel into inversion without moving the leg.  Is excessive inversion of the subtalar joint seen using minimal force? The sole of the foot or visualisation of the neck of the talus should show movement of 45° inwards, the lateral head of the talus will be very prominent. | YES | NO | YES | NO |
| **MIDTARSAL JOINT INVERSION** The patient is supine with their feet over the end of the couch; the midtarsal joint is isolated from the subtalar joint; the forefoot is grasped from lateral to medial along the metatarsals; only minimal-moderate force is applied to invert the midtarsal joint.  Does the midtarsal joint invert beyond 45° so that the plantar surface of the metatarsal heads can be brought inwards by 45 degrees? | YES | NO | YES | NO |
| **MIDTARSAL JOINT AB/ADDUCTION AND DORSI/PLANTARFLEXION** The patient is supine with their feet over the end of the couch; the examiner grasps and stabilises the rearfoot; the forefoot is moved in the direction of ab/adduction and dorsi/plantarflexion. Normal movement should be 1cm in each direction. With minimal force, does the forefoot move easily, almost “wobbling”, in an increased amount? Excessive movement in either of the two planes is a positive result. | YES | NO | YES | NO |
| **METATARSOPHALANGEAL MOVEMENT** The patient is supine with their feet over the end of the couch; the hallux is dorsiflexed using minimal-moderate force.  Does the hallux dorsiflex easily beyond 90° relative to the metatarsal? | YES | NO | YES | NO |
| **EXCESSIVE SUBTALAR MOVEMENT** The patient is to march on the spot and stop on command; the patient is asked to invert their foot and hold the position close to subtalar joint neutral; the patient is then asked to relax their foot; the movement is observed.  Does the arch lower and flatten fully, excessively and easily, with the talus bulging medially? The pronation noted should be at the end of range of the subtalar joint motion so that no further pronation is possible. | YES | NO | YES | NO |
| To score, each limb is calculated separately giving a left score and right score. Each YES is given one mark. A total score of 12 marks is available for each limb, with a score out of 24. The mark is then averaged for a total score out of 12 | Total: score out of 12 | | | |
