# Supplementary material for: Identifying lower limb specific and generalised joint hypermobility in adults: validation of the Lower Limb Assessment Score
Source: BMC Musculoskelet Disord. 2017 Dec 6;18:514. doi: 10.1186/s12891-017-1875-8 (PMC5719901; doi:10.1186/s12891-017-1875-8)
Supplement: Supplementary file 2 — The Beighton Score. This file contains the standardised criteria of the five tests involved in scoring of the Beighton in this study. (DOCX 19 kb) [file 12891_2017_1875_MOESM2_ESM.docx]

# **ADDITIONAL FILE 2**

The Beighton Score [3]

| **Test** | **Score Allocation** |
| --- | --- |
| 1. Passive dorsiflexion of the little fingers beyond 90° | Right: 1  Left: 1 |
| 1. Passive apposition of the thumb to the ventral aspect of the forearm | Right: 1  Left: 1 |
| 1. Hyperextension of the elbow joint beyond 10° | Right: 1  Left: 1 |
| 1. Hyperextension of the knee joint beyond 10° | Right: 1  Left: 1 |
| 1. Placing the palms of the hands flat on the floor while maintaining the knees in full extension | 1 |
|  | Total: score out of 9 |
